# The Spread of Antibiotic Resistance in Natural Environments

**DOI:** 10.3390/antibiotics15090937

**Published:** 2026-09-20

**Authors:** Daniel Gyamfi Amoako

**Affiliations:** 1 Antimicrobial Research Unit, University of KwaZulu-Natal, Durban 4000, South Africa; amoakodg@gmail.com; 2 Department of Pathobiology, University of Guelph, Guelph, ON N1G 2W1, Canada

Antibiotics can be administered to individual patients or animals, but their evolutionary consequences extend across ecosystems. Antimicrobial residues, resistant bacteria, antibiotic resistance genes (ARGs), and mobile genetic elements released from healthcare facilities, households, farms, and pharmaceutical production enter connected environmental pathways that link human, animal, and ecosystem health [1,2,3,4]. Wastewater, surface waters, soils, air, food-production systems, and wildlife-associated environments may act as reservoirs and routes of dissemination. Selective pressures from antimicrobials and co-selective agents can favour the persistence and enrichment of resistance, while diverse microbial communities provide opportunities for horizontal gene transfer [1,2]. The environment is therefore more than a recipient of waste: it is an evolutionary setting in which resistance can persist, change, and circulate across the One Health continuum [2,3,4]. This Special Issue brings together eight contributions from 50 authors across four continents and examines the ecological, genomic, and public health dimensions of environmental AMR.

Shouqair et al. used source-resolved shotgun metagenomics to compare community sewer nodes, hospital outflows, and wastewater-treatment-plant influent and effluent in Dubai. Hospital wastewater was enriched in clinically important class D beta-lactamases, whereas community wastewater and plant influent shared prominent macrolide- and aminoglycoside-resistance genes. Treatment reduced bacterial diversity, but ARG diversity was not significantly lower in effluent, and resistance determinants remained detectable. Virulence functions also shifted toward traits associated with biofilm formation and persistence. These findings show why plant influent alone cannot represent the distinct risks carried by hospital and community wastewater.

Park et al. complemented this metagenomic evidence with a culture-based analysis of 804 Enterococcus isolates from the influent and effluent of 11 wastewater-treatment plants in South Korea. Overall, resistance and biofilm formation did not differ significantly between stages, but individual phenotypes and genes followed different trajectories. Resistance to ampicillin, ciprofloxacin, and gentamicin, combined with *tetM* and *qnrS*, were increased in effluent, whereas other resistance phenotypes and optrA declined. Treatment performance therefore cannot be inferred from a single aggregate measure or from microbial reduction alone.

Mendoza-Guido et al. examined enterobacteria along a pollution gradient in a tropical river. Isolates from the most contaminated site carried more ARGs and plasmids and exhibited broader resistance. One *Escherichia coli* isolate was resistant to seven antibiotics and harboured a plasmid containing 12 ARGs that was closely related to an *IncH* plasmid found in *Salmonella enterica*. The study shows how phenotyping, environmental gradients, whole-genome sequencing, and plasmid analysis can jointly reveal pathways of transferable resistance.

Nnah et al. assessed the virulomes of multidrug-resistant *E. coli* groups representing human, animal, and environmental sources. The three sectors shared a substantial core virulome containing determinants for adhesion, serum resistance, iron acquisition, and stress adaptation. Environmental groups contained the most extraintestinal pathogenic *E. coli*-associated determinants, whereas diarrhoeagenic markers were most frequent in animal groups. The consistently high predicted pathogenicity indicates that resistance surveillance should include virulence monitoring. Isolate-resolved and long-read analyses are now needed to determine whether resistance, virulence, and mobility determinants occur in the same organisms or genetic elements.

The three reviews place these findings within broader environmental pathways. Sassi et al. examine water, soil, and air as reservoirs and transmission routes, emphasizing pharmaceutical discharge, agricultural runoff, sewage, airborne emissions, biofilms, and horizontal gene transfer. They also examine metals and disinfectants as co-selective agents and microplastics as surfaces that may promote persistence and gene exchange. Meradji et al. focus on water systems, tracing connections created by hospital and municipal effluent, stormwater, agricultural runoff, water reuse, and irrigation return flows. Ifedinezi et al. link environmental AMR to food safety and public health and emphasize stewardship, surveillance, regulation, and One Health coordination. Together, the reviews locate AMR control within pollution prevention, sanitation, water treatment and reuse, agriculture, and food-chain protection.

Yuan et al. extend the discussion through the One Earth–One Health framework. Their communication places AMR within ecosystem self-regulation and reconstruction and proposes a dual-mutation concept and mathematical model that combine evolutionary and niche-construction perspectives. The framework recognizes that resistance predates modern antibiotic use and argues that control measures should account for ecological processes that generate, disseminate, and remove resistant organisms and genes. Its proposed mechanisms remain to be tested empirically, but the contribution broadens the discussion beyond the detection of individual determinants.

Several priorities emerge when the contributions are considered together. Environmental surveillance should be organized around transmission pathways, using source-resolved, longitudinal sampling to determine where resistance enters, persists, or becomes enriched. Harmonized metadata, quality controls, analytical pipelines, and reporting standards are essential for comparing these patterns across sites and over time. Treatment performance must then be judged by risk reduction rather than by bacterial removal or gene detection alone. This requires measurements of absolute abundance and viability alongside community composition, resistance phenotypes, and mobile genetic elements. The analytical methods should serve complementary purposes: metagenomics defines resistome diversity; culture and phenotyping confirm viable resistance; long-read sequencing and plasmid reconstruction link determinants to their hosts and genetic contexts; and transfer experiments test their mobility. Parallel measurements of antibiotics, metals, biocides, and other contaminants can help distinguish selection pressure from simple co-occurrence with pollution. These findings should inform risk assessments that integrate pathogenicity, mobility, environmental persistence, exposure route and dose, and available treatment options. This evidence chain can establish whether interventions reduce viable resistant organisms and transferable ARGs under operational conditions. Building it requires coordination across health, agriculture, environmental management, engineering, food safety, and regulation.

Overall, the environment emerges as a major component of AMR transmission. The central task is to connect source attribution and genomic evidence with treatment performance and credible exposure pathways, enabling surveillance to identify where transmission can be interrupted rather than simply where resistance is present. Embedding this evidence within One Health policy is essential to protect human, animal, and ecosystem health.
